# Perceived Cognitive Ability in Lesbian, Gay, Bisexual, and Heterosexual Vietnam Era Veterans

**DOI:** 10.1093/geroni/igaa057.997

**Published:** 2020-12-16

**Authors:** Michelle Hilgeman, John Blosnich, Yasmin Cypel, Fatema Akhtar, Aaron Schneiderman, Erick Ishii, Dennis Fried, Victoria Davey

**Affiliations:** 1 VA Medical Center--Tuscaloosa, Tuscaloosa, Alabama, United States; 2 University of Southern California, Los Angeles, California, United States; 3 U.S. Department of Veterans Affairs, Washington DC, District of Columbia, United States; 4 U.S. Department of Veterans Affairs, Washington, District of Columbia, United States; 5 U.S. Department of Veterans Affaris, Washington, District of Columbia, United States; 6 Department of Veterans Affairs (PQ), Washington DC, United States; 7 VA New Jersey HCS, EAST ORANGE, New Jersey, United States

## Abstract

Lesbian, gay, and bisexual (LGB) Veterans report stress (e.g., discrimination under Don’t Ask Don’t Tell policies) and mental health conditions (e.g., depression) that may increase risk for neurocognitive changes like dementia. Subjective cognitive decline (SCD) can be an early indicator of neurocognitive change – yet no known studies have examined SCD in LGB Veterans. Cross-sectional data from the Vietnam Era Health Retrospective Observational Study (VE-HEROeS) were examined for 260 LGB and 17,796 heterosexual Veterans. VE-HEROeS is the latest probability-based survey of Vietnam Era Veterans (1961–1975) as older adults (2016-2017). SCD was assessed using two subscales of the Functional Assessment of Cancer Therapy-Cognitive Instrument Version-3 (FACT-Cog). Good reliability was observed in this sample: Cronbach’s alpha =.94 for the 7-item Perceived Cognitive Abilities subscale and .88 for the 4-item Comments from Others. Analyses were weighted to account for the complex survey design. LGB Veterans were slightly younger (M=68.3, range 59-84) than heterosexual Veterans (M=69.1, range 58-99, p=.03); were more likely to be female (13% vs 3%, p<.01); and had fewer people living in the household (M=1.7 vs. M=2.1, p<.01). LGB Veterans were also more likely than heterosexual Veterans to report feeling depressed most or all of the time over the past 30 days (5.7% vs. 3.6%, respectively, p<0.01) on a single 5-point Likert-scale. SCD indicators did not vary by Veteran sexual orientation (M=19.69 and M=19.69; M=14.2 and M=14.1) and were elevated compared to published studies in healthy adult samples. More work is needed to examine neurocognitive risk factors in aging LGB Veterans.

